# Real life patterns of care and progression free survival in metastatic renal cell carcinoma patients: retrospective analysis of cross-sectional data

**DOI:** 10.1186/s12885-018-4117-z

**Published:** 2018-02-21

**Authors:** Rana Maroun, Laura Mitrofan, Laure Benjamin, Gaelle Nachbaur, Franck Maunoury, Philippe Le Jeunne, Isabelle Durand-Zaleski

**Affiliations:** 1 GlaxoSmithKline, Health Economics and Outcomes Research, Rueil Malmaison, France; 2 INSERM, ECEVE, UMR 1123, Paris, France; 3 IQVIA, La Défense, France; 4 Statesia, Le Mans, France; 5 URC-ECO, APHP, Paris, France

**Keywords:** Kidney cancer, Real world data, Treatment patterns, Progression free survival, Targeted therapies

## Abstract

**Background:**

Patient characteristics and survival outcomes in randomized trials may be different from those in real-life clinical practice. The objective of this study was to describe treatment pathways, safety, drug costs and survival in patients with metastatic Renal Cell Carcinoma (mRCC) in a real world setting.

**Methods:**

A retrospective analysis was performed using IQVIA real world oncology cross-sectional survey data, a retrospective treatment database collecting anonymized patient-level data in Europe. Data on treatment naïve patients with mRCC who received a first-line targeted therapy in France were extracted for the period 2005–2015. Descriptive analyses were performed on treatment patterns, patient characteristics and safety profiles. Progression Free Survival (PFS) was determined using Kaplan-Meier survival analysis.

**Results:**

One thousand three hundred thirty-one patients with mRCC who received a first-line targeted therapy were included. The male/female sex ratio was 2.5 and 66% of patients were aged > 60 years. 83% of patients had clear cell adenocarcinoma. 83% of patients underwent a surgical procedure, 10% had radiotherapy. In patients who received a first-line targeted therapy, 73% received sunitinib. The mean time from diagnosis to first-line treatment by targeted therapies in patients initially diagnosed with metastatic disease was 3.3 months [95% CI:2.5–4.1]. In patients who received second-line targeted therapy *n* = 257 (19%), the most frequently observed treatment sequences were sunitinib-everolimus (33%) and sunitinib-sorafenib (27%). Adverse events data were available for 501 patients and adverse events were documented in 70% of patients, most frequently diarrhoea. The overall median PFS was 13 months [95% CI:11.5–16].

**Conclusion:**

Patient characteristics were consistent with the literature. Treatment patterns appeared to follow current practice guidelines. Despite some variations, PFS in our study seems to be consistent with findings from other real world studies. Nevertheless, PFS results were higher than those observed in clinical trials. Due to the use of cross-sectional data, PFS in our study should be interpreted with caution.

**Electronic supplementary material:**

The online version of this article (10.1186/s12885-018-4117-z) contains supplementary material, which is available to authorized users.

## Background

The International Society for Pharmacoeconomics and Outcomes Research (ISPOR) has underlined the importance of Real World Data (RWD) to inform decision-making with respect to coverage for health technologies [1]. One reason is that extrapolation of clinical trial results to an entire patient population fails to take into account potential differences between in-study and general patient populations [2]. In addition, RWD are important to understand treatment pathways. In the last decade, there has been extensive recourse to real world studies for the evaluation of treatment patterns and their outcomes, although methodology and findings have varied considerably between studies.

A systematic review compared UK RWD to trial data for patients with metastatic Renal Cell Carcinoma (mRCC) taking sunitinib and reported lower Overall Survival (OS) but similar Progression Free Survival (PFS). The authors explained the poorer OS in the real world setting by a lack of second line treatment and failure to take into account patient prognostic characteristics [3]. Another study estimated PFS in mRCC patients treated by targeted therapies and concluded that even though real world PFS seemed to be consistent with trial-based PFS, practice variation was evident [4]. In contrast, a retrospective study conducted in patients with mRCC reported differences in PFS and adverse events between trials and real world setting [5].

Over the last decade, targeted therapies including Tyrosine Kinase Inhibitors (TKI) and inhibitors of the mammalian Target of Rapamycin (mTOR) have become the standard of care for mRCC patients and have significantly improved prognosis and treatment outcomes [6]. With an increasing number of targeted therapies available for mRCC, understanding treatment patterns and real life outcomes of targeted therapies is important especially because in France their use and outcomes in clinical practice are poorly characterized. Providing such real world evidence could be useful for implementing health economic models and to ensuring patient access to appropriate innovative treatments.

Our objective was to describe real world treatment patterns and outcomes in patients with mRCC including survival, adverse events, drug costs, use of medical technologies and treatment sequences in France.

## Methods

This retrospective analysis used IQVIA-RWD Oncology Cross-Sectional Survey Data (IQVIA-RWD), a cancer treatment database collecting anonymized patient-level oncology data in Europe.

IQVIA-RWD provides retrospective information on patient characteristics and treatment history from the day the physician completes the case report form until diagnosis. Data are collected on a quarterly basis via a web survey methodology in which each physician provides case histories for at least 15 consecutive patients they personally treat. The cap of included patients per quarter, is related to the physician’s specialty and is statistically determined. Physicians complete the case report form using the patient’s medical record. In this manner approximately 2% to 4% of the treated prevalence across cancer types is captured [7]. To ensure that IQVIA-RWD is representative of oncology practice in France, around 210 physicians with different specialties (radiotherapy, dermatology, otolaryngology, gastroenterology, general surgery, gynaecology, haematology, oncology, pulmonology and urology) working in urban and rural regions and in public and private hospitals are included per quarter [8]. To ensure the liability of the collected data, projection techniques are applied and each quarter the data are validated using external sources such as incidence and prevalence data from Globocan and Eucan, cancer registries and published literature. The data that support the findings of this study were available as a ready to use excel spreadsheet. In addition to standard data quality control techniques performed by IQVIA on a regular basis, we have checked data consistency and applied predefined criteria to account for inconsistent values as filled by the physicians.

Study data are available from IQVIA but restrictions apply to the availability of these data, which were used under license for the current study. Consequently, individual patient data cannot be made publicly available. However, researchers can acquire the data set from IQVIA against fees upon request and after signing the data sharing agreement.

For the purpose of this study, we had permission from IQVIA to access the dataset. Data collected in France between October 2005 and September 2015 for patients diagnosed with RCC were extracted. The principal population of interest was patients with metastatic disease treated by a first-line targeted therapy. Targeted therapies included in the analysis were those recommended for the treatment of mRCC and included sunitinib, pazopanib, temsirolimus, everolimus, bevacizumab, axitinib and sorafenib. Included patients were naïve to anticancer systemic treatments (immunotherapy, cytotoxic chemotherapy and targeted therapies). All analyses in this manuscript are conducted on French patients with mRCC who received a first-line treatment by targeted therapies. Progression was defined as treatment cessation due to disease progression (local or distant); patients who stopped treatment due to other reasons were censored. Progression-free survival was estimated only for the first-line. Only adverse events recorded in the patient’s medical record are reported in the database. Dosing information was collected for current treatments and in patients who received a first-line targeted therapy. Drug cost per day was obtained by multiplying the mean cost per mg by the daily dose. Cost data were presented by line of treatment.

### Statistical analyses

Statistical analyses were conducted using SAS 9.3 and R 3.2.3 softwares. Categorical variables are presented as proportions and continuous data as means, medians and standard deviations were reported for continuous variables. PFS was determined using Kaplan-Meier actuarial survival analysis.

## Results

### Patient characteristics

A total of 2527 patients with RCC were identified of whom 1662 (66%) had metastatic disease (Additional file 1: Figure S1). 1331 patients received a first-line targeted therapy. The characteristics of these patients are presented in Additional file 1: Table S2. The sex-ratio was 2.5 M/F and 66% of patients were > 60 years old. The cancer type was clear cell adenocarcinoma (CCA) in 83% of cases and 941 (71%) patients presented metastatic disease at the time of diagnosis. For the 390 patients who were not initially diagnosed with metastatic disease, the mean interval between diagnosis and metastasis was 41 months [95% CI: 37–44]. Moreover, 70% of patients developed metastases in at least two sites. The most frequent sites of metastases were lung (*n* = 942; 71%), lymph node (*n* = 647; 49%), bone (*n* = 404; 30%), liver (*n* = 389; 29%) and brain (*n* = 84; 6%). 612 (46%) patients had at least one associated comorbidity, notably diabetes (*n* = 234), chronic obstructive pulmonary disease (*n* = 130), renal comorbidities (*n* = 73) and cardiac comorbidities (*n* = 65). In the overall cohort, 905 (68%) patients had undergone nephrectomy (Additional file 1: Table S2).

### Patterns of care and treatment costs

**Fig. 1:**
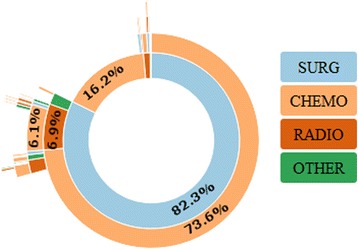
Sunburst diagram of patterns of care in mRCC. The sunburst diagram outlines patterns of care in patients with mRCC. The diagram is read from inside out; for example, 6% of patients had undergone surgery (SURG) followed by radiotherapy (RADIO) followed by anticancer drugs (CHEMO). Percentages are only presented for the most frequent sequences

In the overall cohort, 1108 (83%) patients underwent surgery. Radiotherapy was prescribed in 137 (10%) of patients. Of the 941 patients who had metastatic disease at diagnosis, 528 (56%) underwent nephrectomy. Patterns of care for patients with mRCC are shown in Fig. 1.

The mean time from diagnosis to first-line targeted therapy in patients initially diagnosed with metastatic disease was 3.3 months [95% CI: 2.5–4.1] and the median time was one month [Min-Max: 0–201]. Sunitinib was the most frequently prescribed first-line targeted therapy (*n* = 977; 73%), followed by temsirolimus based regimens (*n* = 180; 13%) and bevacizumab based regimens (*n* = 81; 6%).

Most frequently patients were assigned to the Eastern Cooperative Oncology Group (ECOG) Grades 0 (18%), 1 (59%), 2 (19%), 3 (3%) and 4 (< 1%); the ECOG was not specified in 4% of cases.

Dose and cost analyses were conducted for 1294 patients who received targeted therapy and for whom dose information was available. Table 1 presents the average cost per day, treatment duration and dose per day.

**Table 1:** Summary of real world drug doses and costs by line of treatment

	Mean price (€/mg)	N		Treatment duration (days)	Dose mg/day^a^	Treatment cost (€/day^b^)
*First line*						
Sunitinib	3.52	766	Mean (SD)	71(99)	48(7)	168(27)
			Median [Range]	28[0.23–1043]	50[13–100]	176[6–352]
Sorafenib	0.14	48	Mean (SD)	43(42)	388(151)	54(21)
			Median [Range]	31[1–182]	400[200–800]	56[28–112]
Pazopanib	0.12	19	Mean (SD)	88(87)	716(167)	86(20)
			Median [Range]	49[7–308]	800[400–800]	96[48–96]
Bevacizumab	2.43	52	Mean (SD)	6(10)	706(224)	1684(578)
			Median [Range]	3[1–52]	720[10–1245]	1725[24–3025]
Everolimus	16.19	10	Mean (SD)	86(143)	11(3)	178(51)
			Median [Range]	28[7–455]	10[10–20]	162[162–324]
Temsirolimus	24.26	156	Mean (SD)	9(12)	26(5)	562(192)
			Median [Range]	6[1–77]	25[15–57]	607[87–1379]
*Second line*						
Sunitinib	3.52	23	Mean (SD)	61(41)	51(23)	180(80)
			Median [Range]	28[28–140]	50[25–150]	176[88–528]
Sorafenib	0.14	61	Mean (SD)	73(86)	626(310)	88(43)
			Median [Range]	56[7–623]	400[200–1600]	56[28–224]
Axitinib	13.51	18	Mean (SD)	58(53)	11(3)	149(47)
			Median [Range]	49[7–238]	10[5–20]	135[68–270]
Bevacizumab	2.43	4	Mean (SD)	7(7)	745(209)	1810(508)
			Median [Range]	4[2–17]	745[490–1000]	1810 [1191–2430]
Everolimus	16.19	72	Mean (SD)	118(142)	10(1)	158(19)
			Median [Range]	66[14–784]	10[5–10]	162[81–162]
Temsirolimus	24.26	16	Mean (SD)	9(8)	25(0)	607(0)
			Median [Range]	7[1–29]	25[25–25]	607[607–607]
*Third line*						
Sunitinib	3.52	2	Mean (SD)	70(59)	50(0)	176(0)
			Median [Range]	70[28–112]	50[50–50]	176[176–176]
Sorafenib	0.14	14	Mean (SD)	87(109)	671(347)	94(49)
			Median [Range]	56[14–441]	800[200–1600]	112[28–224]
Axitinib	13.51	3	Mean (SD)	93(89)	12(8)	158(103)
			Median [Range]	49[35–196]	10[5–20]	135[68–270]
Bevacizumab	2.43	3	Mean (SD)	9(7)	850(140)	2066(340)
			Median [Range]	6[4–17]	850[710–990]	2066 [1725–2405]
Everolimus	16.19	13	Mean (SD)	74(115)	10(0)	162(0)
			Median [Range]	28[6–427]	10[10–10]	162[162–162]
Temsirolimus	24.26	12	Mean (SD)	11(10)	27(7)	613(240)
			Median [Range]	8[2–31]	25(25–50]	606[87–1213]

^a^Duration does not include off treatment periods

^b^Dose per day during which treatment is administered

Patients who received two of any drugs listed above as part of current therapy or had more than one completed treatments are counted twice.

Dose information was missing for 27 patients of the 1331 on first-line targeted therapy and odd dose quantities (10 patients) were not taken into account.

### Patterns of targeted therapy and safety data

**Fig. 2:**
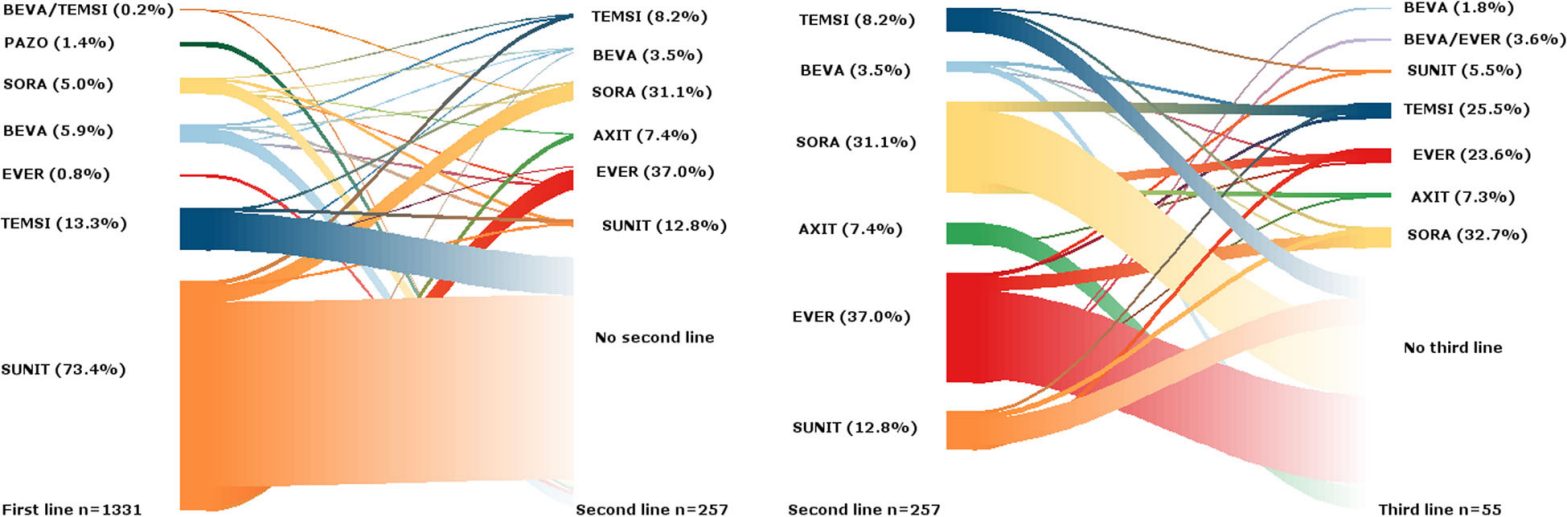
Riverplot showing treatment sequences. The riverplot outlines sequences of targeted therapies. The width of the bar is proportional to the frequency of each sequence. Molecules can be distinguished by colour. In this diagram treatment lines are defined by a change of molecule. BEVA: bevacizumab; SUNI: sunitinib; TEMS: temsirolimus; AXIT: axitinib; SORA: sorafenib; and EVER: everolimus. Percentages are presented by line of treatment and only for treated patients

In patients treated by a first-line targeted therapy (*n* = 1331), TKI, mTOR inhibitors and a combination of both were prescribed at first-line respectively in 1140 (86%), 188 (14%) and 3 (< 1%) patients. In patients who received first-line TKI, 977 (86%) received sunitinib, 78 (6%) bevacizumab, 66 (5%) sorafenib and pazopanib 19 (1%). 177 (93%) patients with an mTOR inhibitor at first-line received temsirolimus and 11 (6%) everolimus. Respectively 19% and 4% had second and third line treatments. Of the 257 patients who had a second-line treatment, the most frequently observed targeted therapy sequences for the first two lines of treatment were sunitinib-everolimus (33%) and sunitinib-sorafenib (27%). The most frequently observed sequence for the first three lines of treatment was sunitinib-everolimus-sorafenib (20%, *n* = 55) (Fig. 2).

Of these 1331 patients, data regarding adverse events with first-line treatment was available for 715 patients. Of those, 501 (70%) patients had at least one adverse event, with multiple events documented in 58% of patients. The most frequent events were diarrhoea (*n* = 162; 23%), anorexia (*n* = 140; 20%), Mucositis (*n* = 108; 15%) and hand foot syndrome (*n* = 93; 13%). The most frequent events were diarrhoea (25%), anorexia (22%), Mucositis (15%) and hand foot syndrome (15%) for sunitinib, fever (17%), diarrhoea (14%) and hand and foot syndrome (14%) for bevacizumab, Mucositis (18%), anorexia (14%), anaemia (13%), stomatitis (13%) and diarrhoea (13%) for temsirolimus. Adverse events by molecule are described in Additional file 1: Table S3.

### Real world progression free survival

**Fig. 3:**
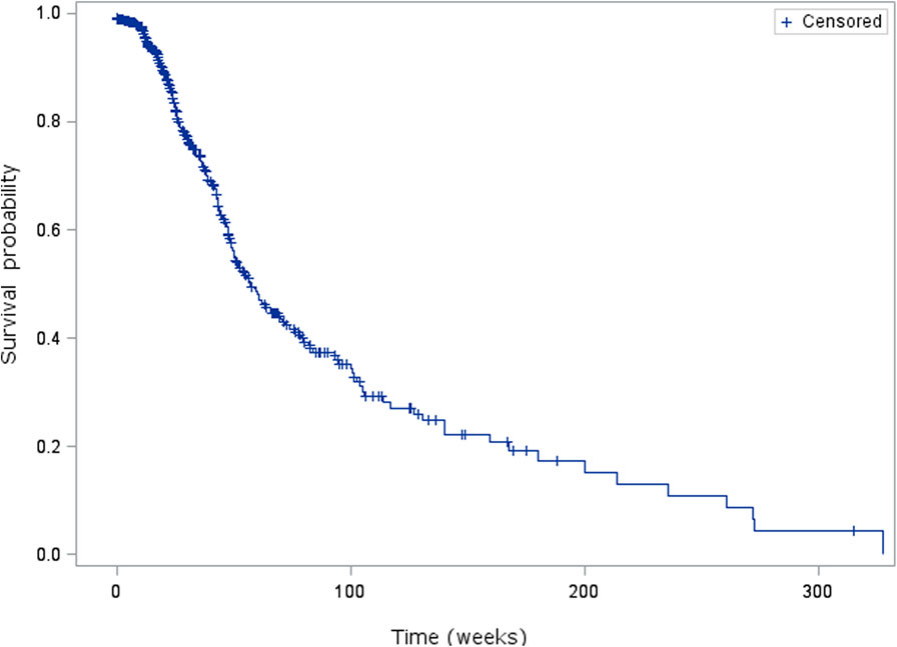
Progression-free survival of patients who received first-line targeted therapy (*n* = 1326)

Median PFS was 13 months [95% CI: 11.5–16] (Fig. 3). The median first-line PFS in patients treated by sunitinib compared to other first-line targeted therapies was 13.7 months [95% CI: 11.5–17.4] versus 11.2 months [95% CI: 9.6–16.5].

## Discussion

This study assessed real world treatment patterns and outcomes in patients with mRCC. Patient characteristics in terms of age, sex and histology are consistent with the known disease epidemiology [9, 10]. Nevertheless, the proportion of patients initially diagnosed with metastatic disease is higher than previously published figures. This difference might be explained by the fact that we worked on a sample constituted exclusively of patients with metastatic disease whereas published figures concern all patients with renal cell carcinoma [11, 12].

A targeted therapy was prescribed to 83% of mRCC patients, most frequently sunitinib (73%). In the overall population, only 19% and 4% of patients had second and third line treatments, respectively. The most frequent sequence of targeted therapies for the first two lines was sunitinib-everolimus. This is consistent with the guidelines of the European Association of Urology and the availability of the treatments through the observed period [13]. First-line treatments seem to be similar between countries and studies [14]. However, there is a slight variability in the use of second and third line treatments between countries and studies. Indeed, our results differ from the results of an American study that described treatment patterns of targeted therapies for mRCC prescribed by community oncologists, in which the most frequently observed treatment sequence was sunitinib-everolimus-bevacizumab [14]. Two different studies, one conducted in the United States and one in Australia, showed that sunitinib and temsirolimus were the most prescribed second-line treatments [4, 15]. These differences can be explained by the availability of treatments at the time of the study. In patients initially diagnosed with mRCC, the mean time to first-line targeted therapy was 3.3 months [95% CI: 2.5–4.1]. These results are consistent with previously published studies in mRCC [16]. Observed daily doses sometimes differed from the recommended dose. For example, the median daily dose of sorafenib at first-line was 400 mg whereas the recommended dose is 800 mg. This might be explained by the safety profile of treatments and related dose reductions.

Documented adverse events for each molecule were consistent with the findings of clinical trials in terms of type of adverse events. Nevertheless, incidence rates were lower than those reported from clinical trials and expanded access studies. For example, diarrhoea was reported in 23% of patients receiving sunitinib and 14% of those treated with sorafenib which is lower than rates observed in clinical trials and expanded access studies [17–19]. Similarly, fatigue has commonly been reported in clinical trials and other real world studies of targeted therapies, but was not reported in this study [18–20]. These differences may result from how adverse events were documented in different types of studies. In addition, since adverse events were not collected for 46% of patients rates of adverse events reported in our study might be underestimated.

Unadjusted PFS in naïve patients receiving a first-line targeted therapy was 13 months [95% CI: 11.5–16.0]. This PFS corresponds to patients treated with any first-line targeted therapy whereas most published results from clinical trials deal with PFS for a single agent. In the case of sunitinib, the observed PFS is slightly higher than PFS published from clinical trials and other real world studies [3, 21, 22]. The first-line PFS of sunitinib in our study is slightly higher than that observed in a joint community-academic retrospective mRCC registry analysis (8.9 months [95% CI: 7.5–10.5]) and lower than that observed in a retrospective analysis of medical records in the United States (15.3 months [95% CI: 5.3–19.5]) [4, 5]. In our study, PFS was not adjusted for the baseline characteristics of the patient and the definition of progression was at the discretion of the physician and may thus have differed between physicians; in contrast, PFS estimates in clinical trials use a standard definition and cases undergo central review. Since in real world practice, clinical assessments to determine progression are not as frequent as in clinical trials, the PFS in our study might be overestimated as compared to PFS determined in clinical trials. Furthermore, there is no longitudinal follow-up for patients in the database; therefore, the database does not adequately convey the subsequent progress of the disease. This might lead to an overestimation of the PFS. It would have been interesting to compare PFS and safety between treatment groups, but such comparisons were not performed due to small sample sizes and the uncontrolled nature of the study.

Our results paint a broad picture of the management of mRCC but there are challenges related to the use of RWD. Although our results are consistent with published studies, they should be interpreted with caution. Since we used a cross-sectional database, the follow-up duration between patients was variable and the sample size for some agents limited. In addition, sequences of targeted therapies should be interpreted in light of the date of marketing authorization for each molecule compared to the date of the study.

Although a certain number of studies aimed at describing real life patterns and outcomes in mRCC patients treated by targeted therapies, none of those studies reflects the French clinical practice and most of these studies focused on a single agent. Hence, information regarding practice patterns and real life outcomes for patients with metastatic renal cell carcinoma treated by targeted therapies is scare in France. This retrospective review of a sizable cohort of mRCC patients treated in real-life practice in France, represents the management of mRCC and real life outcomes of targeted therapies in French settings.

## Conclusion

We described real world PFS, adverse events and treatment patterns in patients with mRCC receiving first-line targeted therapies in France. We noted that real world outcomes and treatment patterns seem to be consistent with data from clinical trials and with practice guidelines. Nevertheless, variability between study results exists, which can be explained in part by differences in modalities of data collection.

## Additional file

Additional file 1: **Figure S1.** Flow chart of included patients. **Table S2.** Table resuming patients ‘characteristics. **Table S3.** Table resuming adverse event data by first-line treatment. (DOCX 37 kb)

## Abbreviations

CCA: Clear Cell Adenocarcinoma
ECOG: Eastern Cooperative Oncology Group
ISPOR: International Society for Pharmacoeconomics and Outcomes Research
mRCC: metastatic Renal Cell Carcinoma
mTOR: mammalian Target of Rapamycin
OS: Overall Survival
PFS: Progression Free Survival
RWD: Real World Data
TKI: Tyrosine Kinase Inhibitors
