# Supplementary material for: Real life patterns of care and progression free survival in metastatic renal cell carcinoma patients: retrospective analysis of cross-sectional data
Source: BMC Cancer. 2018 Feb 21;18:214. doi: 10.1186/s12885-018-4117-z (PMC5822611; doi:10.1186/s12885-018-4117-z)
Supplement: Supplementary file 1 — Figure S1. Flow chart of included patients. Table S2. Table resuming patients ‘characteristics. Table S3. Table resuming adverse event data by first-line treatment. (DOCX 37 kb) [file 12885_2018_4117_MOESM1_ESM.docx]

2,527 patients

with kidney cancer

1,662 patients

with metastatic disease

164 patients

without anticancer drugs

1,498 patients

receiving anticancer drugs*

1,383 patients

received a targeted therapy

1,331 patients received a first-line anticancer treatment by a targeted therapy **

S1 : Flow chart of included patients

* Anticancer drugs includes targeted therapies, cytotoxic chemotherapy and immunochemotherapy

**Only patients who received a first-line systemic anticancer treatment by a targeted therapy were included in the analysis

S 2: Patient characteristics

| **Male *N (%)*** | 941 (70.7) |
| --- | --- |
| **Female *N (%)*** | 390 (29.3) |
| **Age *N (%)*** |  |
| ≤55 | 219 (16.4) |
| 56-60 | 240 (18.0) |
| 61-65 | 279 (21.0) |
| 66-70 | 226 (17.0) |
| 71-75 | 186 (14.0) |
| 76-80 | 106 (8.0) |
| >80 | 46 (3.4) |
| NS | 29 (2.2) |
| **Histology *N (%)*** |  |
| Clear cell adenocarcinoma | 1,108 (83.2) |
| Adenocarcinoma NOS | 109 (8.2) |
| Other | 114 (8.6) |
| **Stage at diagnosis *N (%)*** |  |
| I | 29 (2.2) |
| II | 317 (23.8) |
| III | 0 (0.0) |
| IV | 941 (70.7) |
| NS | 44 (3.3) |
| **Metastatic sites *N (%)**** |  |
| Lung | 942 (70.7) |
| Lymph node | 647 (48.6) |
| Bone | 404 (30.3) |
| Liver | 389 (29.2) |
| Brain | 84 (6.3) |
| Renal | 18 (1.4) |
| Other | 160 (12.0) |
| NS | 4 (0.3) |
| **Comorbidities *N (%)**** |  |
| None | 659 (49.5) |
| Diabetes | 234 (17.6) |
| COPD | 130 (9.8) |
| Renal | 73 (5.5) |
| Cardiac | 65 (5.0) |
| Other | 282 (21.2) |
| NS | 60 (4.5) |
| **Nephrectomy *N (%)*** |  |
| Yes | 905 (68.0) |
| No | 426 (32.0) |

*Percentages may exceed 100% since patients can present with one or more metastasis sites. NS: Not specified, NOS: Not Otherwise Specified, COPD: chronic obstructive pulmonary disease.

**S 3: Adverse events in patients with mRCC receiving first-line targeted therapy**

| **Adverse events** | **Cohort ** N=715** | **Sunitinib N=556** | **Bevacizumab based regimens* N=29** | **Temsirolimus N=87** | **Sorafenib N=15** | **Pazopanib N=19** | **Everolimus N=9** |
| --- | --- | --- | --- | --- | --- | --- | --- |
| Any adverse event | 501 (70.1%) | 411 (73.9%) | 21 (72.4%) | 43 (49.4%) | 12 (80.0%) | 9 (47.4%) | 5 (55.6%) |
| Diarrhoea | 162 (22.6%) | 142 (25.5%) | 4 (13.8%) | 11(12.6%) | 2 (13.3%) | 2 (10.5%) | 1 (11.1%) |
| Anorexia | 140 (19.6%) | 122 (21.9%) | 3 (10.3%) | 12 (13.8%) | 2 (13.3%) | 1 (5.3%) | 0 (0.0%) |
| Mucositis | 108 (15.1%) | 82 (14.7%) | 2 (6.9%) | 16 (18.4%) | 1 (6.7%) | 3 (15.8%) | 4 (44.4%) |
| Hand foot syndrome | 93 (13.0%) | 84 (15.1%) | 4 (13.8%) | 1 (1.1%) | 4 (26.7%) | 0 (0.0%) | 0 (0.0%) |
| Anaemia | 87 (12.2%) | 74 (13.3%) | 0 (0.0 %) | 11 (12.6%) | 1 (6.7%) | 0 (0.0%) | 1 (11.1%) |
| Stomatitis | 81 (11.3%) | 65 (11.7%) | 3 (10.3 %) | 11 (12.6%) | 2 (13.3%) | 0 (0.0%) | 0 (0.0%) |
| Nausea and vomiting | 74 (10.3%) | 66 (11.9%) | 2 (6.9%) | 2 (2.3%) | 1 (6.7%) | 1 5.3%) | 2 (22.2%) |
| Rash | 61 (8.5%) | 52 (9.4%) | 2 (6.9%) | 4 (4.6%) | 2 (13.3%) | 0 (0.0%) | 1 (11.1%) |
| Neutropenia | 54 (7.5%) | 48 (8.6%) | 2 (6.9%) | 4 (4.6%) | 0 (0.0%) | 0 (0.0%) | 0 (0.0%) |
| Cachexia | 23 (3.2%) | 21 (3.8%) | 0 (0.0 %) | 2 (2.3%) | 0 (0.0%) | 0 (0.0%) | 0 (0.0%) |
| Thrombocytopenia | 23 (3.2%) | 20 (3.6%) | 1 (3.4%) | 2 (2.3%) | 0 (0.0%) | 0 (0.0%) | 0 (0.0%) |
| Fever | 19 (2.7%) | 13 (2.3 %) | 6 (20.7%) | 0 (0.0%) | 0 (0.0%) | 0 (0.0%) | 0 (0.0%) |
| Hair loss | 9 (1.2%) | 6 (1.1%) | 1 (3.4 %) | 0 (0.0%) | 2 (13.3%) | 0 (0.0%) | 0 (0.0%) |
| Infection | 6 (0.8%) | 5 (0.9%) | 1 (3.4 %) | 0 (0.0%) | 0 (0.0%) | 0 (0.0%) | 0 (0.0%) |
| Other | 100 (14.0%) | 75 (13.5%) | 7 (24.1%) | 7 (8.0%) | 4 (26.7%) | 6 (31.6%) | 1 (11.1%) |

* One patient had a Bevacizumab/Temsirolimus regimen

**Adverse events (AE) were not reported for 616 patients due to the modalities of collecting AE. Indeed, QuintilesIMS database relies exclusively on the usage of secondary data and no primary data is generated or collected. Hence AE data were not collected during some specific time periods. Indeed, in the article we have included data over a 10 years’ period, and the questionnaire had modifications over time and one of these modifications was the inclusion of AE question with pre-defined answer possibilities.
